# Pharmacist management of atrial fibrillation in UK primary care: a qualitative study

**DOI:** 10.1186/s40545-022-00486-0

**Published:** 2022-12-09

**Authors:** Shahd Al-Arkee, Julie Mason, Antje Lindenmeyer, Zahraa Jalal

**Affiliations:** Institute of Clinical Sciences, University of Birmingham, Birmingham, United Kingdom

**Keywords:** Pharmacists, Management, Atrial fibrillation, Primary care, Qualitative study

## Abstract

**Background:**

Atrial fibrillation (AF) affects up to 2% of the UK population. AF is a potentially long-term condition that needs management, and as such primary care pharmacists may have a substantial role in supporting the management of AF.

**Objectives:**

This study aims to explore the role of primary care pharmacists, working in community pharmacies and general practices (GPs), in supporting the management of AF. Furthermore, this study investigates pharmacists’ confidence in their knowledge and their attitudes towards incorporating AF-associated mobile apps use into their current practice.

**Methods:**

A qualitative study was conducted, using one-to-one semi-structured, audio-recorded interviews with primary care pharmacists. The topic guide was developed based on pharmacy visits and included the most relevant constructs from the ‘consolidated framework for implementation research (CFIR)’. All interviews were audio-recorded, transcribed verbatim and thematically analysed until saturation was achieved, guided by Braun and Clarke’s 6-step research method. This study was given a favourable opinion on 5 September 2019 by the University of Birmingham (UOB) Research Ethics Committee (Reference ERN_19-0908).

**Results:**

Thematic saturation was achieved after 11 interviews with primary care pharmacists (seven community pharmacists, and four GP pharmacists). Three main themes emerged relating to (1) the clinical role of pharmacists in the management of AF; (2) knowledge and awareness; and (3) prioritisation of resources. The first highlighted that primary care pharmacists were an underutilised resource within AF management. The second demonstrated that pharmacists, especially those based in the community, felt a lack of confidence in their knowledge of AF and its management, mainly community pharmacists due to other roles taking precedence over clinical roles. Both community and GP pharmacists expressed the need to have further training in this therapeutic area to be able to effectively support patients with AF. The third shed light on the pharmacists’ views relating to the technological revolution in healthcare. Pharmacists expressed an interest in using apps to support their current practice.

**Conclusions:**

Primary care pharmacists supported an extended care to AF management from screening to consultations, yet the provision of such services remains limited and inconsistent. Future research should focus on understanding the ways in which pharmacists’ role can be adapted toward greater involvement in clinical care.

**Supplementary Information:**

The online version contains supplementary material available at 10.1186/s40545-022-00486-0.

## Background

Atrial fibrillation (AF) is the most commonly diagnosed cardiac arrhythmia. In the UK, approximately 2% of the population have already been diagnosed with AF and a sustained increase in prevalence is predicted in the future [1]. The increase in AF prevalence may be attributable to better detection [2–4], alongside increasing age and predisposing conditions [5]. AF is independently associated with a twofold increased risk of all-cause mortality in women and a 1.5-fold increase in men [6–8]. AF is also associated with a fivefold increased risk of stroke [9], with an increased risk of dementia and heart attacks [10, 11]. AF is potentially a long-term condition. As a result, AF has been a focus of many UK government initiatives including the National Health Service’s (NHS) long-term plan, which, in part, sets out goals to prevent 150,000 strokes, heart attacks, and dementia cases by the year 2029 [12].

Primary care pharmacists, i.e. those working in community pharmacies and general practices (GPs) may have a substantial role in supporting the NHS long-term plan. UK primary care systems are being reshaped and pharmacy services reappraised to cope with growing demand. Models of care are continually evolving to better utilise the clinical skills and knowledge of pharmacists; one such model that the NHS has supported is for community pharmacists to move from a predominantly medicines supply-focused role towards a clinical role [13]. Community pharmacists started to deliver advanced services, e.g., medicine use reviews (MURs) in 2005 and new medicine services (NMS) in 2011, which are intended to improve patients’ knowledge, medication adherence, and medicines optimisation [13]. Further, the Department of Health and Social Care in England included community pharmacists as a provider of the NHS Health Checks, this program is designed to assess an individual’s risk of developing long-term conditions and targets the entire population aged 40–74 years [14]. Another model that has emerged is the integration of pharmacists within general practices (GPs) [15]. GP pharmacists support patients with medication-related issues; and recently, structured medication reviews (SMRs) have been introduced, which are designed to include a comprehensive clinical review of patients’ medicines [16].

Thus far, evidence suggests that both community and GP pharmacists may play a significant role in reducing medical risk factors associated with cardiovascular diseases (CVD) events [17] and improving medication adherence [18]. However, a recent multinational study raised concerns that pharmacists did not always feel confident in their knowledge to support patients on anticoagulants, and highlighted the need to invest in education to address pharmacists knowledge gaps and enable them to confidently support patients in this therapeutic area [19]. The use of technology, including mobile applications (apps), has been suggested as one way to overcome a shortfall in pharmacists’ knowledge [20].

The role of primary care pharmacists in supporting long-term conditions management has been explored in previous qualitative studies, for example, their importance in supporting medication adherence and for the provision of lifestyle advice [21–23]. However, there is a paucity of published evidence regarding pharmacists’ role specifically in the management of AF. This study aims to explore the role of primary care pharmacists working in community and GP in supporting the management of AF. Furthermore, this study investigates primary care pharmacists’ confidence in their knowledge, and their attitudes towards incorporating AF-associated mobile apps use into their current practice.

## Methods

### Ethical approval

This study was approved by the University of Birmingham (UOB) Research Ethics Committee (Reference ERN_19-0908) on 5 September 2019. All methods were performed in accordance with the University of Birmingham Code of Practice for Research. Informed consent was obtained from every participant. Before signing the consent form, participants received information that described the nature and purpose of the study.

### Study design

Semi-structured, qualitative interviews were conducted with UK-based registered pharmacists employed within primary care settings, i.e. community pharmacies and general practices (GPs). This method was chosen because its interactive nature allows generation of rich data on the topic discussed [24]. Participants were purposively sampled (i.e. those working in community pharmacies and GPs, and conducted clinical consultations in their daily practice) and recruited through professional connections.

To inform the interview topic guide, three preliminary visits were made to community pharmacies by the main researcher (SA) to understand about workflow; handing out of medication; the role of pharmacy support-staff; and pre-structured patient–pharmacist consultations and their duration. During these visits, informal discussions were held with pharmacists about the use of apps and digital technology. The topic guide was developed to include the most relevant constructs from the ‘consolidated framework for implementation research (CFIR)’ [25] (Additional file 2). The CFIR was chosen as it can guide formative evaluations of complex interventions (such as pharmacist management of AF); it provides a pragmatic structure that embraces and unifies key constructs from published implementation theories. Broadly, this framework consists of five domains: the first relates to the characteristic of the intervention itself; the next two centre on the outer and inner context; the fourth focusses on the individuals involved in the intervention, and the fifth cuts across all other domains to consider intervention implementation.

A semi-structured pilot interview was conducted and after a review of the data generated and topic guide, researchers agreed to include the pilot data in the final analysis as no fundamental amendments to the topic guide were required and the data were highly relevant to the research question; this method was supported by Holloway as a basic concept for qualitative research [26].

### Data collection

Pharmacists were invited to participate in one-to-one semi-structured, face-to-face, audio-recorded interviews. Following written informed consent, interviews were undertaken by the main researcher (SA). The interviews, lasting 34–40 min, were conducted in the period from September 2019 to March 2020 at the University of Birmingham.

### Data analysis

Thematic analysis was guided by Braun and Clarke’s 6-step method [27]. Qualitative data analysis with an inductive approach was conducted while the initial phases of data collection were in progress, so that emergent results could be incorporated into subsequent qualitative data collection. All of the audio-recorded interviews were transcribed verbatim by the main researcher (SA) and ambiguities resolved through matching transcripts with original recordings by another researcher (JM). The data were imported into NVivo qualitative data analysis software (QSR International Pty Ltd. Version 12. released in 2020) [28] for the purpose of coding and thematic analysis. Analysis involved independent reading and rereading the transcribed data by two researchers (SA and ZJ) to identify common codes and themes with interpretations verified by a third researcher (JM). A minimum of 8 interviews was specified for thematic analysis and saturation was set as the point at which no new ideas emerged from 3 further consecutive interviews in accordance with Francis et al. 2010 [29]. The data were reviewed by a separate qualitative researcher (AL) for consistency. The emergent themes were presented to all researchers for discussion and validation. Statistical analysis of the demographic data was performed using Statistical Package for the Social Sciences (SPSS) 27.0 (IBM Corp., Armonk, N.Y., USA).

### Reporting

The study is reported in accordance with the ‘consolidated criteria for reporting qualitative research (COREQ)’ checklist [30] (Additional file 1).

## Results

### Demographic data

Thematic saturation was achieved after 11 one-to-one semi-structured interviews with pharmacists; 7 were from community pharmacies, and 4 were from GP practices. Participants’ length of professional experience ranged from 8 to 27 years (mean 17.9, median 13.0 years). Further participant demographics can be seen in Table 1.

**Table 1:** Demographic characteristics of the primary care pharmacists (n = 11)

Characteristics	Participants % (n)
*Gender*	
Male	72.7 (8)
Female	27.3 (3)
*Pharmacy sector*	
Community	9.1 (1)
General practice	9.1 (1)
Academia and community	54.5 (6)
Academia and general practice	18.2 (2)
Academia, community, and general practice	9.1% (1)
*Prescriber status*	
Independent prescriber (IP)	54.5 (6)
*Specialist area*	
Cardiovascular disease	27.3 (3)
Diabetes	9.1 (1)
Hypertension	9.1 (1)
Mental health	9.1 (1)
*Qualifications post-IP registration*	
Diploma	36.4 (4)
Masters	18.2 (2)
Non-prescriber	45.5 (5)
*Post-graduate (PG) qualifications*	
None	27.3 (3)
Doctorate	18.2 (2)
*Participation in PG consultation skills courses*	81.8 (9)

### Themes

Three main themes emerged from the data, these themes related to (1) the clinical role of pharmacists in the management of AF; (2) knowledge and awareness; and (3) prioritisation of resources. These three themes, subthemes, and codes can be seen in Table 2.

**Table 2:** Classification of the thematic codes

Themes and subthemes	Codes
Theme 1. Clinical role of pharmacists	
Subtheme. Identifying patients with AF	Pharmacist–patient interactions—daily basis
	Identification—discharge letter
	Identification—dispensed medication
	Identification—patient medical records (PMR)
Subtheme. Performing roles beyond the focus on medication	Counselling—nothing upon handing out the medication
	Counselling—with pre-structured consultations
	Counselling—no specific services target AF
	Screening—no services/in isolated incidents
	Screening—opportunistically performed
Subtheme. Conducting comprehensive AF consultations	Consultations form—face-to-face consultations or by telephone
	Consultations strategies—patient-centred approach
	Consultations strategies—patient-initiated questions
	Consultations strategies—build rapport
	Consultations’ aim—medication adherence
	Consultations’ aim—medication reconciliation
	Consultations’ aim—information provision
	Consultation aids—pre-set interview schedule
	Consultation aids—digital proforma specific AF
Subtheme. Dealing with workflow	Time pressure—expanded responsibilities
	Consultations length—variation in length
	Consultations length—business requirements
Subtheme. Conducting consultations within professional boundaries	Boundary—lack interprofessional collaboration
	Boundary—lack multidisciplinary teamwork
	Boundary—lack shared decision-making use
	Boundary—lack access to full patients’ data
	Documentation—on the computer-based system
Subtheme. Changes in prescribing practices and medicines preference over time	Prescribing—DOACs patient/HCPs preference
	Prescribing—directed by secondary care
	Prescribing—same agent of DOACs
	Perception/patients—warfarin is risk medication
	Perception/patients—no leeway with DOACs
Theme 2. Knowledge and awareness	
Subtheme. Lacking specific guiding information and starving for knowledge	Adequacy of training—basic information on AF
	Adequacy of training—pharmacists’ confidence
	Adequacy of training—interest in further training
	Available resources—healthcare website
	Available resources—BNF / NICE guidelines
	Available resources—manufacturer information
	Available resources—multiple mobile apps
	Access to resources—good / available online
Theme 3. Prioritisation of resources	
Subtheme. Interest in using technology and mobile apps in practice	Interest in internet-based technology
	Technological revolution—world is digitalised
	Apps-based technology—range of functionality
	Apps functionality—reminders
	Apps functionality—education
	Apps functionality—self-management
	Apps disadvantages—concerns / elderly
	Apps disadvantages—concerns / training
	Apps disadvantages—concerns / time
	Apps disadvantages—concerns / funds

### Theme 1: clinical role of pharmacists

#### Identifying patients with AF

The first subtheme related to identification of patients with AF. Both community and GP pharmacists confirmed daily interactions with patients with AF. While community pharmacists reported the use of discharge letters and prescriptions to identify patients with AF, GP pharmacists reported using patient medical records (PMR).

*“Just looking at their PMR patient medication records… I do work in the medical practice; I don’t work in the community pharmacy anymore. Now we have access to the records in more detail so that’s where we know that’s atrial fibrillation” *(Participant 7, GP pharmacist).

The majority of community pharmacists reported that they identified patients with AF through assumptions about prescribed medication; as the same prescribed medication could be used for conditions rather than AF (e.g., anticoagulants).

*“Well, I don’t know, hundred percent know, it is just from medication that I am making an assumption”* (Participant 6, Community pharmacist).

### Performing roles beyond the focus on medication

The second subtheme focused on the range of clinical services offered in practice. Community pharmacists did not perform counselling upon handing out medication. The main reason provided for this was that medication handover was performed by pharmacy support-staff at busy times. Thus, community pharmacists lost the opportunity to speak to patients at that point.

*“The only time I do counsel on this when I am doing a medicine use review, or a new medicine service. Generally, in terms of routine handing out, it is not usually done by me, it is done by other staff and the chance to counsel is obviously gone at that point. So, no”* (Participant 6, Community pharmacist).

Community pharmacists reported that AF counselling was performed within pre-structured/clinical consultations for medicine optimisation such as MURs, and the NMS, while GP pharmacists performed SMRs. Both community and GP pharmacists reported that their consultations were part of long-term conditions management, and there were no specific/entire services that target patients with AF.

*“There is nothing to do specifically with atrial fibrillation, but obviously as part of something called medicines use review, we will discuss their medication at that point”* (Participant 4, Community pharmacist).

Other services were related to screening of patients for asymptomatic AF ‘silent AF’ or ‘subclinical AF’. Both community and GP pharmacists confirmed that, services for screening and detection of AF were non-existent other than in isolated incidents and research contexts. However, community pharmacists opportunistically identified patients with a potential diagnosis of AF through NHS health checks, e.g., blood pressure, or pulse rate; whereas GP pharmacists used the quality improvement tool ‘Guidance on Risk Assessment in Stroke Prevention for Atrial Fibrillation (GRASP-AF)’ to generate a list of patients requiring a review for AF diagnosis. Both community and GP pharmacists reported that they had not used any technology-based devices such as iPhone ECG to screen for AF but expressed a desire to use them as they may be more confirmatory than conventional pulse checks.

*“I would like to be able to have access to technology to use it because that would be a lot more confirmatory to use something like for example their devices AliveCor, Kardia Mobile”* (Participant 9, GP pharmacist).

Furthermore, GP pharmacists reported that community pharmacists could provide substantial support in screening for AF and alerting the GPs by utilising the existing primary care infrastructure.

*“I think community pharmacy should be able to highlight patient who are potentially have atrial fibrillation and then flag that for the GP that would be very beneficial… If they sent us a form saying, you know, we have highlighted these patients, we think it might be AF, please could you review them”* (Participant 9, GP pharmacist).

### Conducting comprehensive AF consultations

The third subtheme centred on pharmacists’ clinical consultations. Several elements were highlighted. The consultations were conducted either face-to-face or by telephone as required as part of advanced services and approved by the NHS. Community and GP pharmacists confirmed awareness of the importance of communicative strategies to perform effective consultations for patients with AF. Ultimately, the majority felt that they had built trusting relationships with patients at the professional level.

*“I think I do build rapport but then again I’ve been working in my area for 20 years, I’ve been working in the same sort of the areas 20 years… I think it develops because that the communication skills and the fact you are looking at it from shared perspectives, the clinician telling the patients what they need to do, it is looking what it is appropriate, sharing that decision”* (Participant 8, GP pharmacist).

Community and GP pharmacists were fully conscious of the importance of patient-centred approaches to perform effective consultations. They reported that they tended to deliver the consultations by adapting the questions of the interview schedules which were provided by national guidance to shape their consultations, as they aimed to achieve naturally flowing conversations which allowed consultations to be tailored to patients’ needs. Furthermore, GP pharmacists reported that they used digital proforma specific for AF as a guide to facilitate AF consultations. This proforma is a decision support tool designed to assist in prescribing anticoagulation therapy for the prevention of stroke in patients with AF.

*“Yes, I do actually have a proforma that I do, basically there is on the internet, there is the Keele AF anticoagulation website and on there, I used that as my guide actually”* (Participant 9, GP pharmacist).

The other important elements were related to pharmacists’ perceptions of the consultations’ aims. Community and GP pharmacists believed that the pre-structured consultations were ways to support patients with AF by tackling medication issues and providing further information. Community pharmacists mainly focused on medication adherence assessment, while GP pharmacists did medicines reconciliation, and raised patients’ awareness by the acronym ‘FAST’ (Face, Arm, Speech, Time) warning signs to identify stroke early.

*“To be honest, the very main point of MUR and NMS and the questions you would ask is, how are you taking this medication? and how often do you take it? do you think you are missing any doses? …”* (Participant 1, Community pharmacist).

### Dealing with workflow

This subtheme considered the time-pressure and workflow surrounding consultations. Community pharmacists reported being heavily involved in the dispensing process. Alongside this, some reported that they also provided other clinical services such as vaccination clinics. Thus, pharmacists felt that this had potentially placed pressure on them and influenced consultation standards, because they lacked flexibility to conduct consultations according to specific patient needs. The views of GP pharmacists were different from those of community pharmacists; GP pharmacists were being able to spend dedicated time within the GP environment.

*“Yeah, so that’s the difficulty is the time management. So, we do a lot of services, so we have a diary system and it’s really busy. We do a lot of vaccinations, so it’s a bit hit and miss as to, who you can fit in, when”* (Participant 3, Community pharmacist).

Community pharmacists suggested that the length of their consultations ranged from 10 to 15 min compared to GP pharmacists which were approximately 20 to 30 min. Community pharmacists reported that although they wanted to spend more time in consultations, this was difficult as consultation duration was directed by business requirements, such as set targets for the number of consultations conducted annually. However, GP pharmacists did not mention this as an issue with their consultations.

*“I do think that could be better but if I would spend 45 min doing an NMS and MUR, I am pretty sure my boss would say what you are doing spent that long”* (Participant 6, Community pharmacist).

Community pharmacists reported that they received professional support from other pharmacy staff in enabling them to carry out their pharmacy services as much as possible. When dealing with patients in the consultation rooms, the staff used standard operating procedures (SOPs) to enable smooth running of the pharmacy.

*“When I am in consultation room, I’m relying on my staff to give out prescriptions, but if there’s anything I’m concerned about, so for example, if it’s somebody on warfarin and they know, they will know what they need to ask that patients. We have SOPs”* (Participant 3, Community pharmacist).

### Conducting consultations within professional boundaries

This subtheme considered interprofessional relations that promote collaborative healthcare. Community pharmacists reported that they did not regularly network with other healthcare professionals (HCPs) in external organisations. They described that they only shared the outcomes of consultations when there were concerns and this was by letter or email to the relevant general practitioner.

*“We would only share the outcomes if there is an issue, so when there is an issue there is a specific letter, we generate from the Patient Medication Record system to the GP”* (Participant 4, Community pharmacist).

Community and GP pharmacists informed that they routinely entered consultation outcomes into local computer-based systems following their local procedures. However, these systems were set up differently in community when compared to GP. GP pharmacists reported these systems to be accessible for all other HCPs within the same practice, which allowed them to work as multidisciplinary healthcare teams. This was not the case for community pharmacists as their local computer-based systems were not linked to external organisations. Furthermore, when pharmacists were asked about the access to patients’ data, community pharmacists reported that they had no access to NHS records ‘patient medical records (PMR)’, as their companies were privately owned businesses and contracted to the NHS to provide the pharmacy services. Whereas GP pharmacists confirmed that they had access to full patients’ data ‘PMR’, and these were essential for performing effective consultations for patients with AF.

*“Do you feel that having access to full patients’ data would help you to manage your consultations effectively?”* (Researcher).

*“Absolutely, because without a blood test, for example, we wouldn’t be able to see if this is appropriate for this patient to start on the anticoagulant, renal function as well things like that”* (Participant 9, GP pharmacist).

When community and GP pharmacists were asked about shared decision-making approaches, community pharmacists reported that they did use these approaches in supporting medication adherence. However, because prescribing was not part of their role, they did not use shared decision-making approaches around medication choice as these consultations had already been conducted when they saw the patients, i.e. the decision had been already made by prescribers. This was regardless of whether they had undertaken the independent prescribing course or not. However, GP pharmacists who had undertaken the independent prescribing course reported that they offered medication choices, explained risks and benefits, and supported patients in making the choices. They did not guide patients to particular medication, rather they performed an open discussion.

*“To what extent do you use shared decision-making approaches about prescribed medication for patients with AF?*” (Researcher).

*“zero. In community pharmacy, it’s always the decision has been made for us, we are just dispensing”* (Participant 6, Community pharmacist).

*“I give them the options to choose which they would like, explain the risks and the benefits, … so I don’t guide them to any particular anticoagulant, but I do guide them, this is the risks and benefits”* (Participant 9, GP pharmacist).

### Changes in prescribing practices and medicines preference over time

The sixth subtheme considered the changes in general prescribing practice for the management of AF because of the introduction of direct oral anticoagulants (DOACs) in addition to vitamin K antagonist oral anticoagulants (e.g., warfarin). Community and GP pharmacists reported that there was an increase in DOACs prescribing and a consequent decrease in warfarin use over the last five years. They described that the prescription of oral anticoagulants (OACs) in primary care was directed by secondary care resulting in all patients tending to be prescribed the same DOAC (e.g., apixaban).

*“I think nowadays we just tend to stay away from warfarin, so it is about choosing the right DOAC. What in, the place that I work at, all the patients tend to be on the apixaban… I think it’s because it’s what the hospital prefers, and because of that everyone is like pretty much on it”* (Participant 11, GP pharmacist).

Community and GP pharmacists reported that DOACs were used in preference to warfarin and that HCP and patients’ preferences for switching to DOACs were driven by the requirement for fewer clinic visits for international normalised ratio (INR) monitoring and perception of the safety of the therapy.

*“I mean patients do prefer the DOACs in the sense that they don’t have to go for regular INR checks, and things like that… So, monitoring wise it’s a lot better”* (Participant 2, Community pharmacist).

*“I think a lot of doctors now just want to switch patients over to DOACs… So, I think a lot of healthcare professionals want to make that switch now”* (P11, GP) (Participant 11, GP pharmacist).

Community and GP pharmacists stated that the adherence rates to OACs is relatively ‘good’, especially for warfarin. The main reason given for this was an impression that patients’ sense warfarin to be a riskier medicine than a DOAC due to the numerous drug–drug and drug–food interactions and frequent INR monitoring associated with warfarin.

*“It is good. Yeah, probably with warfarin, the most known drug and the risk of bleeding, and the INR monitoring and everything, So, they tend to be a bit more alert and cautious compared to other once”* (Participant 1, Community pharmacist).

Community and GP pharmacists confirmed that they were aware of the risk of missing a dose of DOAC as this may lead to a loss of protection for stroke prevention, but they reported that patients had not often realised that DOACs had a lesser time period of anticoagulation effect.

*“I don’t think they perceive the risk of potential stroke if they miss doses, they have less doses, I think from my experience patients perceive warfarin to be a risky medicine than the DOACs”* (Participant 8, GP pharmacist).

### Theme 2: knowledge and awareness

#### Lacking specific guiding information and starving for knowledge

This subtheme considered the extent of pharmacists’ confidence in their knowledge and skills to support the management of AF. Community pharmacists reported that they had only received basic information on AF and its management as part of their training and that they lost most of their original knowledge over time due to lack of provision within their clinical role. However, GP pharmacists reported that they felt ‘competent’ in conducting AF consultations. Both community and GP pharmacists confirmed the importance of continuing education and professional development to feel more confident in performing effective AF consultations; further, they expressed an interest in having more training and upskilling.

*“I think I’ve lost most of my knowledge with time, because yeah it just the nature of the job, I think for something, because important to exist, if there was an upskilling, absolutely, I’ve been more than willing to do that. Once I’ve lost my knowledge, I don’t think knowledge is up to the level it should be”* (Participant 6, Community pharmacist).

When community and GP pharmacists were asked about resources that they used for information within consultations for patients with AF, they reported that they felt overloaded due to many diverse sources, such as the BNF, recognised healthcare website, NICE guidelines, manufacturer information, and multiple apps. Further, they reported a lack of specific guidance in using information resources for the management of AF.

*“Which resources (if any) do you use when counselling patients with AF?”* (Researcher).

*“well, nothing particular to atrial fibrillation, it will be the BNF, it will be the BNF to start with, if there is anything, you’re not certain about, and you need guideline, you might just go to NICE guideline. But nothing like particular for AF. It is like any other patients on the medication”* (Participant 1, Community pharmacist).

When they were asked about ease of access to resources, all reported ‘good’, due to the information being open, accessible, and available online.

*“How easy is it for you to access information regarding AF and AF management?”* (Researcher).

*“I don’t think there is any problem, everything is available online… yeah, it is easy to access”* (Participant 5, Community pharmacist).

### Theme 3: prioritisation of resources

#### Interest in using technology and mobile apps in practice

This subtheme considered the pharmacists’ views on the technological revolution in healthcare. Community and GP pharmacists reported that they had an interest in the use of digital-based technology, especially apps, as they felt that the systems in the workplace were becoming increasingly smarter and more digital.

*“I would love to keep using technology because I think we need to work smarter not harder”* (Participant 9, GP pharmacist).

Community and GP pharmacists envisaged that healthcare apps could have a range of functionality such as reminders, education, and self-management. They also felt that apps accredited by ‘NHS providers’ or ‘Medicines and Healthcare products Regulatory Agency (MHRA)’ had the potential to be reliable sources of information, as patients were often curious to know more about their health status and tended to consult ‘Google’ even before making a visit to any HCPs.

*“People now try and read more, and they go lot, like to internet and google things. They’re doing that through app filter the information for them, and give them only what is right and trusted to read”* (Participant 1, Community pharmacist).

Despite pharmacists’ positive views about technology, particularly apps, most reported their concerns about elderly patients who may find them hard to use; the need for effective training for pharmacists and patients in the use of apps; and also, a lack of time available for both pharmacists and patients to be able to use these apps and to be able to have training in their use.

*“The only disadvantage is in people are perhaps not able to use the technology very elderly. Not familiar with smartphones”* (Participant 4, Community pharmacist).

*“Time is the only disadvantage, time and training”* (Participant 6, Community pharmacist).

Furthermore, pharmacists expressed concern about a lack of access to technology devices, e.g., ‘tablet computers’, in practice, as employers were not currently providing devices, and pharmacists only have their personal mobile phones, which they cannot use. Community and GP pharmacists would not be able to use healthcare apps and share patient data on their personal mobile phones due to the ‘General Data Protection Regulation’.

*“I can think of … is by data sharing, if people don’t agree to share their data. For example, if it is my personal phone and asking some patients to give me some personal details, they might be reluctant to give their information stored on my phone, I might think and say, that I will delete it, but that’s the only problem they have might be data confidentiality breach”* (Participant 5, Community pharmacist).

Regarding the use of AF-associated apps, most community and GP pharmacists were aware of the existence of certain apps for AF management (e.g., My AF and AF manager), but did not use them in practice. There were several reasons cited for this; these apps were not part of their practice instructions, and also, the ability to use these apps required investment in tablet computers which were not provided.

*“You need to invest basically money in it, and in most sort of sectors you would have to think whether this investment is worth or not…”* (Participant 1, Community pharmacist).

## Discussion

### Principal findings

Three main themes emerged from interviews with community and GP pharmacists about their role in supporting the management of AF within primary care: the clinical role of pharmacists; knowledge and awareness; and prioritisation of resources.

Community and GP pharmacists who participated in this study confirmed that they were not aware of any pharmacist-led services for consulting or screening or detection of AF in the UK, only general consultations, as a part of long-term conditions management. Community and GP pharmacists reported that they attempted to use patient-centred approaches and focused on patients as a whole individual situated within social contexts. However, their aims tended to focus predominantly on adherence and medicines reconciliation. Community pharmacists confirmed that time pressures, business requirements and lack of access to full patient records were often an impediment to effective consultations. This was not the case for GP pharmacists.

A key finding was that pharmacists, especially those based in community lacked confidence in their knowledge on AF and its management. Both community and GP pharmacists expressed a need for more training on AF and its management and a desire to use of AF-associated mobile apps to support AF management in daily practice.

### Strengths and limitations

To the authors’ best knowledge, this is the only qualitative study that has explored the role of UK primary care pharmacists in supporting the management of AF. The observation pharmacy visits enabled a focus on questions about how AF support was being performed and managed alongside the provision of other daily work tasks. A qualitative/semi-structured interview approach was used to achieve comprehensive coverage of the topic. The interviews with both community and GP pharmacists enabled the perceptions of these groups to be represented, thus providing a more holistic view of pharmacists’ role in primary care.

The lack of participation of other stakeholders (e.g., general practitioners and patients with AF) in this study was a limitation. However, previous qualitative studies which have investigated the clinical role of pharmacists in the management of several different long-term conditions in the UK, for example, diabetes [21], and CVD [23], included only the perspectives of pharmacists on their role [21, 23]; a similarity with this qualitative study. The fact that not all the interviewees were qualified as independent prescribers, and those that were specialised in conditions other than AF (e.g., diabetes, and mental health) was another limitation. However, this may strengthen the relevance of the findings because it more accurately reflects the actual practice where only 10% of all UK pharmacists are specialised in CVD as independent prescribers [31]. Given that the majority of interviewees were jointly working in academia and primary care, that was not representative of all pharmacists in the UK, which may be a bias.

### Comparison with existing literature

Previous qualitative studies which have explored the clinical role of pharmacists in the management of several different long-term conditions in the UK, for example, asthma [22], diabetes [21], and CVD [23], have reported promising findings with regard to supporting patient education and self-management [21], medication adherence [22], and providing lifestyle advice [23].

This study found pharmacists to be an underutilised resource within AF management. In addition, pharmacists were not aware of any pharmacy services for AF detection and AF-specific consultations other than in certain isolated incidents. This finding is understandable as the UK national guidance does not support these pharmaceutical services [1, 13, 16, 32]. Similarly, a recent narrative review of many international studies exploring the pharmacist’s role in primary and secondary care, concluded that they are a potentially untapped resource with regard to integrated AF care and suggested pharmacy service frameworks need to be re-structured to support AF-focussed interventions [33].

This study found that AF consultations were only performed as part of long-term condition management for patients on anticoagulants, rather than for patients specifically with AF. Not all patients with AF are treated with anticoagulants [34], and this may be a potential missed opportunity for provision of clinical support. Community pharmacists’ perceptions about the aims of consultations tended to focus on medication adherence. However, as with other qualitative studies [35, 36], this leaves greater scope to provide the patient education and lifestyle advice required by national guidance for community pharmacy services [13]. While this is particularly important to minimise the risk of AF-related adverse events, community pharmacists felt time pressures prohibitive to the conduct of comprehensive consultations. This finding aligns with existing qualitative studies, which report community pharmacists’ existing work obligations could have an effect on the depth of consultations [37, 38]. In contrast, GP pharmacists claimed to integrate patient education into medicines reconciliation focussed consultations. In part, they felt this was due to a less time-pressured environment.

This study also highlighted a lack of pharmacist collaboration and other HCPs such as general practitioners, especially for those based in the community. Previous qualitative studies also report the scarcity of such interprofessional relations [39, 40]. This may not only lead to duplication of work, but also cause confusion and inconvenience for patients with the potential for multiple consultations with different HCPs within the same period. In concordance with a previous observational qualitative study examining the NMS [39], lack of access to full patient records was highlighted as an issue by community pharmacists. In contrast, GP pharmacists felt more integrated with the multidisciplinary healthcare pathway and reported being able to access patient medical records. Thus, time-pressure, professional barriers, and system setup (e.g., lack of access to full patient records) were reflected as issues in performing clinical support in the community.

Pharmacists in this study described how, in their patient consultations, they can play an important role in supporting the use of OACs in the management of AF. The implementation of changes to national and international guidelines for the management of AF, where new patients are now recommended to be started on a DOAC instead of warfarin [41–43], were reflected by pharmacists in this study reporting changes in prescribing practice and a move to DOACs in preference to VKAs. While DOACs were perceived as a more convenient therapy choice by HCPs and patients, pharmacists felt that patients often failed to appreciate the short duration of anticoagulation effect offered by DOACs when compared to warfarin. Similarly, a recent qualitative study indicated that patients had less knowledge about DOACs [44].

To allow community and GP pharmacists to have a more clinical input in advising patients with AF requires them to have optimal knowledge and confidence. This could be achieved with specific training on AF management and prescribing OACs. However, pharmacists in this study, especially those based in the community, reported only basic knowledge on AF and its management. This is supported by a recent educational intervention study, which demonstrated that although pharmacists were knowledgeable on AF management there were areas in which their knowledge could be improved [45]. Further, pharmacists were not aware of any resources specific to the management of AF and consistent with another study [46], used the BNF as a main resource to support AF management. While resources like the BNF are useful, they cover a plethora of diseases, requiring pharmacists to search through that information to find what they need. These findings demonstrate a potential need to improve the knowledge base of pharmacists for AF management. Specific training might be helpful as well as the introduction of concise targeted resources.

AF-associated mobile apps, for both patients and HCPs, have been suggested as a potential resource to support AF management [20, 47]. Pharmacists in this study expressed enthusiasm for this form of technology and felt that apps could potentially facilitate the provision of more effective support for HCPs and patients. Yet, they also conveyed minor concerns about incorporating app use into practice and about using them with elderly patients. Previous studies, however, have shown positive perspectives on apps with this population [48, 49]. Published qualitative studies have also reported that apps could play an important role in screening for AF [50], and monitoring for stroke [51], thereby suggesting that well-designed apps may support more general self-management for patients with AF.

### Implications for research and practice

Community and GP pharmacists provide a wide range of clinical services and are integral to the delivery of the NHS long-term plan [52, 53]. Data presented in this study suggest that, as experts of medicines and healthcare, pharmacists, especially those based in primary care may be well placed to conduct AF screening and AF-specific consultations. It may be expected that the specification of the entire service will develop from the government’s cardiovascular agenda, which aims to improve the detection and management of AF [54].

Evidence shows that community and GP pharmacists can have a clinical role in supporting the detection of the AF [50, 55]. This study suggests that by utilising the strengths of existing practice infrastructure and their clinical expertise, community pharmacists may help in the screening of AF. However, pharmacists perceive that they need a better knowledge of AF and its management. This suggests a need for further training to be consistent with the national vision of the expanded clinical role of pharmacists. Future clinical guidelines may want to consider the favourable profile of apps technology highlighted by community and GP pharmacists in order to support the management of AF. This is in alignment with NHS digital plan, which, identifies apps and other digital tools as a means to improve patient outcomes and services [56].

Future research should focus on assessing the primary care pharmacists’ knowledge of AF and its management, as well as evaluating the effectiveness of using AF-associated mobile apps alongside the pre-structured consultations. Research should also focus on understanding the ways in which time-efficiency can be created for pharmacists to enable them to use apps and perform effective consultations. Further, developing an integrated contractual arrangement between pharmacists, especially those based in the community and other HCPs may be a way to reduce the barriers to interprofessional communication and ensure multidisciplinary working.

## Conclusion

Primary care pharmacists supported AF management by providing a range of extended care from screening to consultations; yet the provision of such services remains limited and inconsistent. Primary care pharmacists perceived that they had a lack of confidence in their knowledge, especially those based in the community, due to lack of provision within their clinical role. Thus, primary care pharmacists expressed an interest in receiving further training on AF and its management and using AF-associated mobile apps in practice to support the management of AF.

## Supplementary Information

**Additional file 1.** Interview Topic Guide.

**Additional file 2. **COREQ Checklist.

## Data Availability

All data generated or analysed during this study are included in this published article [and its additional files]. Supplementary data related to this study can be found at Additional file 1.

## Abbreviations

AF: Atrial Fibrillation
Apps: Mobile Applications
BNF: British National Formulary
CFIR: Consolidated Framework for Implementation Research
COREQ: Consolidated criteria for Reporting Qualitative Research
CVD: Cardiovascular Diseases
DOACs: Direct Oral Anticoagulants
GPs: General Practices
GRASP-AF: Guidance on Risk Assessment in Stroke Prevention for Atrial Fibrillation
HCPs: Healthcare Professionals
IP: Independent Prescriber
MHRA: Medicines and Healthcare products Regulatory Agency
MURs: Medicine Use Reviews
NHS: National Health Service
NICE: National Institute for Health and Care Excellence
NMS: New Medicine Services
OACs: Oral Anticoagulants
PMR: Patient Medical Records
SMRs: Structured Medication Reviews
SOPs: Standard Operating Procedures
UOB: University of Birmingham
