# Supplementary material for: Pharmacist management of atrial fibrillation in UK primary care: a qualitative study
Source: J Pharm Policy Pract. 2022 Dec 9;15:98. doi: 10.1186/s40545-022-00486-0 (PMC9733171; doi:10.1186/s40545-022-00486-0)
Supplement: Supplementary file 1 — Additional file 1. Interview Topic Guide. [file 40545_2022_486_MOESM1_ESM.pdf]

Primary care pharmacists interview topic guide

Section I: Demographic information

1. For record purposes how would you describe your gender? *This information just that we can balance the numbers of our participants from all genders.*
2. I would like to start by asking you to tell me about your career journey as a pharmacist. *Prompts: What year did you first register as a pharmacist in the UK?*
3. Have you spent any time working in other sectors of pharmacy? *Prompts: Could you tell me more about that?*
4. What if any, postgraduate qualifications have you undertaken? *Prompts: What course was it?*
5. Are you a prescriber? *Prompts: If yes, are you an independent or supplementary prescriber? What is your specialist area?*
6. Could you describe any postgraduate courses you have undertaken in order to further develop your consultation skills? *Prompts: Did it lead any further qualifications? Have any of the courses you have taken covered atrial fibrillation (AF) and/or its management?*

Section II: Pharmacy services for patients with AF

7. In your daily practice, do you come across patients with AF? *Prompts: If yes, how do you know they have AF?*
8. What kind of services do you offer which are related to AF? *Prompts: Do you dispense, counsel upon handing out medication, screen for patients with AF?*
9. To screen for patents with AF, how significant would be using the devices/ tools? *Prompts – What are these devices/ tools do use (if any)?*
10. When do you counsel patients that have AF (i.e., when giving out a dispensed prescription, during pre-arranged consultations)? *Prompts: What kind of information do you supply patients?*
11. During the consultations for patients with AF, to what extent do you use the pre-set interview schedule? *Prompts: Are you still using the interview schedule?*
12. When you conduct the consultations for AF patients, what issues do you discuss? *Prompts: To what extent do you use shared decision-making approaches about prescribed medication for patients with AF? Do you discuss stroke and how to recognise signs of stroke 'FAST' (Face, Arm, speech, Time)?*
13. Which resources (if any) do you use when counselling patients with AF? *Prompts: NICE guidelines, any other resources? How easy is it for you to access information?*

14. How do you feel about managing and supporting patients with AF? *Prompts: Do you feel you have adequate skills, knowledge?*
15. To what extent do you use shared decision-making approaches about prescribed medication for patients with AF? *Prompts: Do you see patients more on warfarin or direct-acting oral anticoagulants (DOACs) in consultations?*
16. In your experience, how would you rate adherence to oral anticoagulants? *Prompts: Any* difference between patient's adherence to DOACs, compared to warfarin?
17. Could you describe how you envisage your role (as a primary care pharmacist), in the management of AF patients? *Prompts: How do you envisage your role reflect the reality of your day-to-day practice?*
18. To what extent do you feel you build rapport (a trusting relationship) with your AF patients? *Prompts: For AF patients in particular are there any particular barriers?*
19. How do you document the outcomes of the consultations for AF patients? *Prompts: On a paper-based system? By using the computer-based system?*
20. How do you share the consultations' outcomes of AF patients with other Healthcare Professionals (HCPs)? *Prompts: Any collaboration between pharmacist and GP?*
21. How do you usually manage your consultation regarding time and workflow at your practice? *Prompts: How do you maintain workflow during the consultations?*

Section III: App-based technology in AF management

22. What kind of smartphone or tablet computer apps are used in consultations in general for all patients? *Prompts: What about in patients specifically with AF?*
23. How do you think pharmacists might benefit from using an app as a resource of information in AF management? *Prompts: How do you feel that using an app may influence your consultation with AF patients? Do you envisage any disadvantages?*
24. Following on from the previous question, how do you feel AF patients would benefit from using an app in AF management? *Prompts: Is that regarding improving patients' knowledge of AF? Do you envisage any disadvantages?*
25. How well do you think the use of AF- associated apps (e.g., My AF and AF Manager apps) in practice? Prompts: Do you use these apps in your practice?

Before we finish, is there anything else you would like to share with me about the management of AF patients or the use of apps in healthcare that you think I might find useful? Thank you very much for your time.
